# Low‐Level Viremia Impairs Efficacy of Immune Checkpoint Inhibitors in Unresectable Hepatocellular Carcinoma

**DOI:** 10.1111/liv.70066

**Published:** 2025-03-13

**Authors:** Rong Li, Wenli Li, Qing Yang, Yujuan Guan, Yongru Chen, Peilin Zhu, Kaiyan Su, Qi Li, Xiaoyun Hu, Mengya Zang, Miaoxian Zhao, Manhua Zhong, Jingquan Yan, Keli Yang, Wei Zhu, Zhanzhou Lin, Guosheng Yuan, Jinzhang Chen

**Affiliations:** ^1^ State Key Laboratory of Organ Failure Research, Guangdong Provincial Key Laboratory of Viral Hepatitis Research, Department of Infectious Diseases Nanfang Hospital, Southern Medical University Guangzhou Guangdong People's Republic of China; ^2^ Department of Respiratory Medicine The Third People's Hospital of Chengdu, Affiliated Hospital of Southwest Jiaotong University Chengdu Sichuan People's Republic of China; ^3^ Department of Infectious Diseases Zhuhai People's Hospital Zhuhai Guangdong People's Republic of China; ^4^ Department of Hepatology Guangzhou Eighth People's Hospital Affiliated to Guangzhou Medical University Guangzhou Guangdong People's Republic of China; ^5^ Department of Hepatology Huizhou Central People's Hospital Huizhou Guangdong People's Republic of China

**Keywords:** hepatitis B virus, hepatocellular carcinoma, immune checkpoint inhibitors, low level viremia, maintained virological response

## Abstract

**Background and Aims:**

The impact of low‐level viremia(LLV) on the efficacy of immune checkpoint inhibitors (ICIs) in unresectable hepatocellular carcinoma(uHCC) patients remains unclear. This study aims to investigate the effect of LLV on the outcomes of ICIs‐based therapy in patients with uHCC.

**Methods:**

In this multicenter retrospective study, we included patients with uHCC who received ICIs‐based therapy at four centres between January 2019 and December 2022. All patients were positive for HBsAg and were on nucleos(t)ide analogues (NAs) antiviral therapy. Propensity score matching (PSM) and inverse probability of treatment weighting (IPTW) were used to balance baseline characteristics between the LLV and maintained virological response (MVR) groups. Proteomic analysis was performed on a subset of patients to identify differential protein expression.

**Results:**

A total of 329 patients (mean age 56 years; 92.4% male; 70.8% BCLC stage C) were included, with 170 patients in the LLV group and 159 in the MVR group. The objective response rate (ORR) was significantly lower in the LLV group compared to the MVR group (21.2% vs. 36.5%, *p* = 0.002), as was the disease control rate (DCR) (78.8% vs. 92.5%, *p* < 0.001). Median progression‐free survival (mPFS) was shorter in the LLV group (7.6 vs. 12.6 months, *p* < 0.001), as was median overall survival (mOS) (22.8 vs. 40.0 months, *p* < 0.001). These differences remained consistent after PSM and IPTW adjustments. Multivariate analysis identified LLV as the only independent risk factor for overall survival (hazard ratio [HR] 0.522, 95% CI 0.348–0.781; *p* = 0.002). Proteomic analysis revealed significant differences in the expression of Flt3L, SLAMF1 and FGF‐5 proteins between the LLV and MVR groups.

**Conclusion:**

LLV is associated with poorer responses to ICIs‐based therapy and reduced survival in patients with HBV‐related uHCC.

**Summary**

- Our findings indicate that low‐level viremia (LLV) is associated with poorer responses to immune checkpoint inhibitors‐based therapy and reduced survival in patients with HBV‐related unresectable HCC.
- LLV is an independent risk factor affecting patient survival. Proteomic analysis revealed significant differences in the expression of Flt3L, SLAMF1 and FGF‐5 proteins between the LLV and maintained virological response (MVR) groups.

## 1 Introduction

Liver cancer is the sixth most common malignancy worldwide and the third leading cause of cancer‐related mortality, with hepatocellular carcinoma (HCC) accounting for over 80% of cases [1]. Despite advances in cancer therapy, the efficacy of immune checkpoint inhibitors (ICIs), particularly those targeting the PD‐1/PD‐L1 pathway, remains limited in HCC treatment, with an objective response rate below 20% due to off‐target effects and low response rates [2, 3, 4].

Combination therapies have been explored to overcome these challenges. Anti‐angiogenic agents, for example, can normalise tumour vasculature, facilitating immune cell infiltration, alleviating hypoxia and enhancing the efficacy of ICIs [5, 6]. Additionally, local treatments such as transarterial chemoembolization (TACE), hepatic arterial infusion chemotherapy (HAIC), ablation, and radiotherapy effectively reduce tumour burden and modify the tumour microenvironment (TME), thereby augmenting the antitumour effects of ICIs [7, 8]. Consequently, combining ICIs with molecular targeted therapy (MTT) and local interventions has emerged as a prevalent strategy for managing advanced HCC.

In China, hepatitis B virus (HBV) infection is the leading cause of HCC. Effective long‐term antiviral therapy with nucleos(t)ide analogs (NAs) is critical for slowing the progression of chronic HBV infection and improving patient outcomes [9]. While first‐line NAs achieve complete virologic response in most chronic hepatitis B (CHB) patients, approximately 20% continue to experience low‐level viremia (LLV), defined as detectable HBV DNA levels below 2000 IU/mL [10]. In these patients, LLV is associated with a higher risk of end‐stage liver disease and HCC development. Moreover, among those with HCC, LLV correlates with shorter survival compared to patients maintaining a virological response (MVR) [10, 11, 12, 13].

Despite the known risks of LLV, its impact on the efficacy of ICIs in patients with unresectable HCC (uHCC) remains unexplored. Addressing this gap is crucial, as understanding the influence of LLV on treatment outcomes could inform clinical decision‐making and patient management strategies. Therefore, this study aims to investigate how LLV affects treatment outcomes and survival in HBV‐related HCC patients receiving ICIs‐based therapy. By elucidating the relationship between LLV and ICIs efficacy, we hope to contribute valuable insights that could enhance therapeutic approaches for this patient population.

## 2 Materials and Methods

### 2.1 Patients

This multicenter retrospective study included patients with uHCC who received ICIs‐based therapy at four hospitals: Southern Medical University Southern Hospital, Huizhou Central People's Hospital, Zhuhai People's Hospital, and the Eighth People's Hospital of Guangzhou Medical University, between January 2019 and December 2022. The study was approved by the Southern Hospital Ethics Committee (Approval No. NFEC‐2023‐593).

Inclusion criteria:

1. Age ≥ 18 years, any gender.
2. Pathologically or clinically confirmed BCLC stage B or C HCC.
3. Treatment with ICIs in combination with MTT, with or without local treatments (HAIC, TACE, ablation or radiotherapy).
4. Presence of at least one measurable intrahepatic lesion representing the primary tumour burden.
5. Positive hepatitis B surface antigen (HBsAg) status, with > 1 year continuous NA antiviral therapy and regular HBV DNA monitoring (every 3–6 months).
6. At least one assessment of tumour efficacy.

Exclusion criteria:

1. Pathologically confirmed cholangiocellular carcinoma, mixed hepatocellular carcinoma, sarcomatoid hepatocellular carcinoma, or fibrolamellar carcinoma.
2. Presence of other malignancies.
3. Treatment with non‐standard anti‐tumour drugs (e.g, traditional or proprietary Chinese medicines).
4. Irregular ICIs or antiviral medication use.
5. Inconsistent HBV DNA monitoring.
6. Adjuvant ICI therapy after surgical resection.
7. History of organ or bone marrow transplantation.

### 2.2 Definition of LLV

LLV is defined as detectable HBV DNA levels < 2000 IU/mL in CHB patients who have been consistently receiving entecavir (ETV), tenofovir disoproxil fumarate (TDF), tenofovir alafenamide (TAF) or tenofovir amibufenamide (TMF) for ≥ 48 weeks. Continuous LLV is characterised by stable HBV DNA levels between 20 and 1999 IU/mL throughout the follow‐up, whereas intermittent LLV involves fluctuating levels within this range. MVR is defined as CHB patients undergoing NAs antiviral therapy for at least 48 weeks, with a sustained HBV DNA level maintained below 20 IU/mL.

### 2.3 Liver Assessment

Liver fibrosis and function were assessed using non‐invasive indicators to evaluate disease severity and prognosis. The aspartate aminotransferase to platelet ratio index (APRI) and the fibrosis‐4 (FIB‐4) score were used as fibrosis markers, with APRI > 2 indicating liver fibrosis and FIB‐4 > 3.25 suggesting advanced fibrosis (stage 3 or 4). Higher APRI and FIB‐4 scores are associated with poorer outcomes.

Liver function was further evaluated using the Albumin‐Bilirubin (ALBI) grade and the Indocyanine Green retention rate at 15 min (ICG15). The ALBI grade, derived from serum albumin and bilirubin levels, provides an objective assessment of liver function, while the ICG15 test, measuring retention of indocyanine green dye 15 min after administration, offers additional insights into hepatic reserve and function.

### 2.4 Proteomic Analysis

Proteomic profiling was conducted on serum samples from a subset of 20 patients (10 from each of the LLV and MVR groups) using Olink technology. This analysis aimed to identify inflammation‐related proteins that differed significantly between groups. Gene Ontology (GO) enrichment, KEGG pathway analysis, and Reactome pathway analysis were used to explore the biological pathways associated with differential protein expression.

### 2.5 Outcomes

Progression‐Free Survival (PFS): Time from ICI initiation to tumor progression or death.

Overall Survival (OS): Time from ICI initiation to death from any cause.

### 2.6 Data Collection and Assessments

Baseline data were collected within 1 week prior to ICI treatment initiation, including demographic information, liver function tests, virological parameters, antiviral and anticancer treatment regimens, tumour efficacy, and survival data. Tumour efficacy was assessed using the Response Evaluation Criteria in Solid Tumours (RECIST) version 1.1, with imaging data independently reviewed by two experienced radiologists; a third senior radiologist resolved any discrepancies.

### 2.7 Statistical Analysis

Statistical analyses were conducted using SPSS version 26.0. Continuous variables with a normal distribution are presented as means ± standard deviations, while non‐normally distributed data are shown as medians with interquartile ranges. Group differences were assessed using t‐tests, Mann–Whitney U tests for continuous variables, and *χ* ^2^ or Fisher's exact tests for categorical data. Kaplan–Meier curves were used to analyse PFS and OS, with group differences assessed by the log‐rank test. Propensity score matching (PSM) was applied to reduce potential confounding, using nearest‐neighbour matching with a caliper of 0.02 to achieve a 1:1 ratio. Inverse probability of treatment weighting (IPTW) was applied to balance baseline characteristics across groups. Cox regression analysis identified factors impacting OS, with statistical significance set at *p* < 0.05.

## 3 Results

### 3.1 Patient Characteristics

**FIGURE 1:**
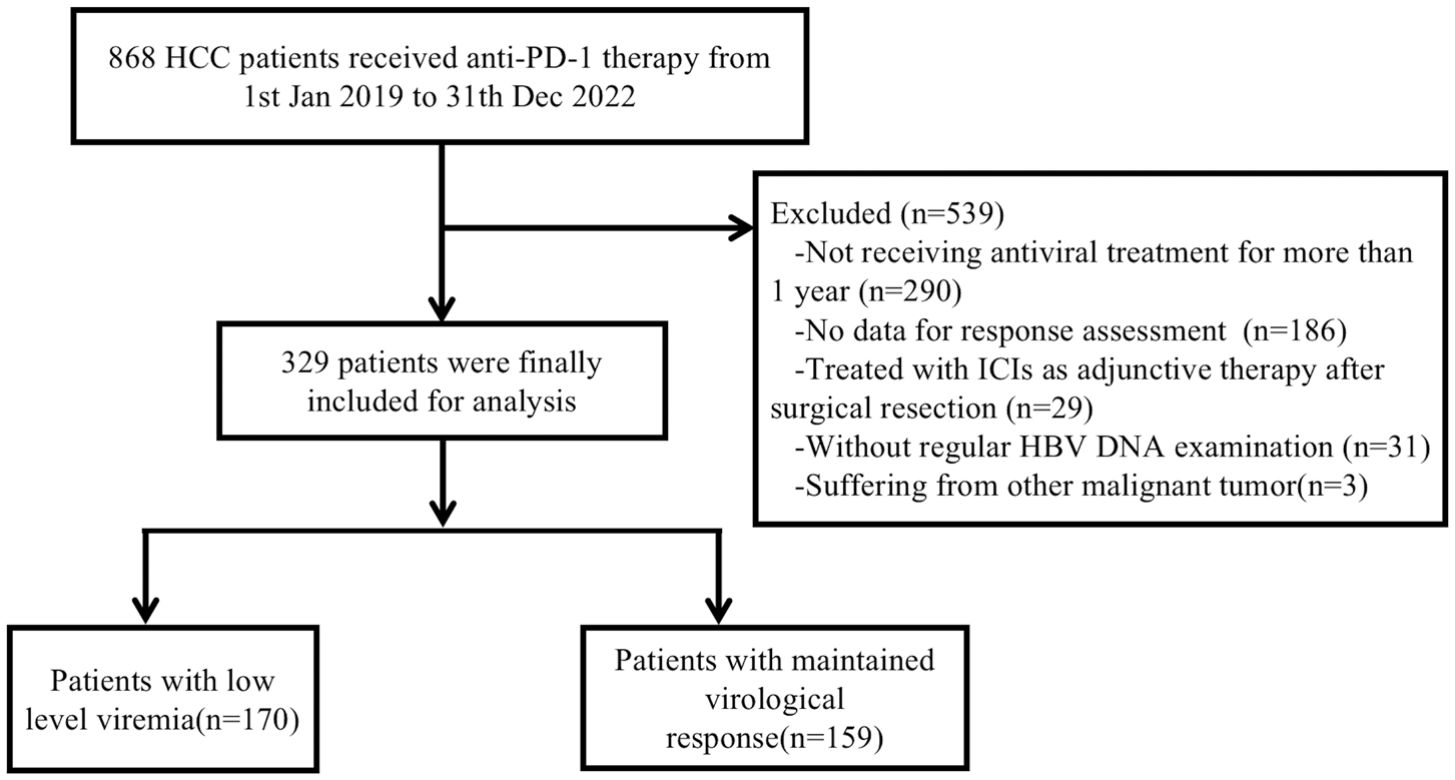
The flowchart of patient selection. HCC, hepatocellular carcinoma; ICIs, immune checkpoint inhibitors.

A total of 329 patients were included in this study, with 159 in the MVR group and 170 in the LLV group (Figure 1). The two groups showed no significant differences in gender, age, ECOG performance status, tumour number, maximum tumour diameter, ascites, liver cirrhosis, Child‐Pugh classification, AFP levels or HBeAg status. However, the LLV group had a later BCLC stage and a higher proportion of patients with extrahepatic metastases. Baseline ALT and HBV DNA levels were also elevated in the LLV group. Specifically, 130 of the LLV patients (76.5%) were at BCLC stage C, compared to 64.8% in the MVR group (*p* = 0.02); similarly, 90 of the LLV patients (52.9%) had extrahepatic metastases versus 40.9% in the MVR group (*p* = 0.029). Both groups had comparable antiviral and antitumour treatment regimens. Following PSM, each group had 124 patients with no significant differences in their baseline characteristics (Table 1). IPTW adjustment further ensured similar baseline characteristics across groups (Table S1; Figure S1).

**TABLE 1:** Baseline patient characteristics.

Characteristics	Before PSM			After PSM		
	MVR	LLV	*p*	MVR	LLV	*p*
Patients	159	170		124	124	
Male sex	147 (92.5)	157 (92.4)	0.973	114 (91.9)	115 (92.7)	1.000
Age ≥ 60 years	65 (40.9)	59 (34.7)	0.248	46 (37.1)	46 (37.1)	1.000
ECOG performance^Δ^						
0	122 (76.7)	122 (71.8)	0.174	92 (74.2)	95 (76.6)	0.759
1	36 (22.6)	42 (24.7)		31 (25.0)	27 (21.8)	
2	1 (0.6)	6 (3.5)		1 (0.8)	2 (1.6)	
Previous treatment						
No	79 (49.7)	84 (49.4)	0.960	61 (49.2)	67 (54.0)	0.497
Yes	80 (50.3)	86 (50.6)		63 (50.8)	57 (46.0)	
BCLC						
B	56 (35.2)	40 (23.5)	0.020	38 (30.6)	37 (29.8)	1.000
C	103 (64.8)	130 (76.5)		86 (69.4)	87 (70.2)	
Extrahepatic metastasis	65 (40.9)	90 (52.9)	0.029	60 (48.4)	53 (42.7)	0.427
Embolus	64 (40.3)	78 (45.9)	0.303	48 (38.7)	53 (42.7)	0.603
Number of tumours ≥ 2	134 (84.3)	137 (80.6)	0.380	101 (81.5)	101 (81.5)	1.000
Tumour diameter, cm						
< 10	128 (80.5)	123 (72.4)	0.082	99 (79.8)	93 (75.0)	0.430
≥ 10	31 (19.5)	47 (27.6)		25 (20.2)	31 (25.0)	
Ascites	39 (24.5)	45 (26.5)	0.686	29 (23.4)	34 (27.4)	0.533
Cirrhosis	126 (79.2)	132 (77.6)	0.725	99 (79.8)	96 (77.4)	0.749
Diabetes	29 (18.2)	29 (17.1)	0.779	23 (18.5)	22 (17.7)	1.000
ALT levels ≥ 40 U/L	51 (32.1)	73 (42.9)	0.042	48 (38.7)	47 (37.9)	1.000
PLT ≥ 100 × 10^9^/L	119 (74.8)	135 (79.4)	0.324	94 (75.8)	96 (77.4)	0.880
Child‐Pugh grade						
A	128 (80.5)	139 (81.8)	0.770	100 (80.6)	100 (80.6)	1.000
B	31 (19.5)	31 (18.2)		24 (19.4)	24 (19.4)	
Serum AFP ≥ 400 ng/mL	53 (33.3)	72 (42.4)	0.092	42 (33.9)	49 (39.5)	0.392
HBV DNA ≥ 2000 IU/mL	26 (16.4)	44 (25.9)	0.035	26 (21.0)	27 (21.8)	1.000
HBeAg positive	17 (10.7)	31 (18.2)	0.053	16 (12.9)	19 (15.3)	0.678
Anti‐viral therapy						
ETV	105 (66.0)	103 (60.6)	0.784	81 (65.3)	72 (58.1)	0.446
TDF	14 (8.8)	17 (10.0)		12 (9.7)	13 (10.5)	
TAF	34 (21.4)	43 (25.3)		26 (21.0)	32 (25.8)	
TMF	6 (3.8)	7 (4.1)		5 (4.0)	7 (5.6)	
Local regional therapy						
None	52 (32.7)	63 (37.1)	0.289	44 (35.5)	45 (36.3)	0.489
HAIC	43 (27.0)	47 (27.6)		36 (29.0)	34 (27.4)	
TACE	41 (25.8)	40 (23.5)		28 (22.6)	30 (24.2)	
Ablation	15 (9.4)	7 (4.1)		12 (9.7)	5 (4.0)	
Radiotherapy	8 (5.0)	13 (7.6)		4 (3.2)	10 (8.1)	
Immune therapy^Δ^						
Camrelizumab	50 (31.4)	52 (30.6)	0.415	35 (28.2)	42 (33.9)	0.543
Sintilimab	25 (15.7)	35 (20.6)		21 (16.9)	27 (21.8)	
Tislelizumab	48 (30.2)	36 (21.2)		37 (29.8)	24 (19.4)	
Atezolizumab	12 (7.5)	16 (9.4)		9 (7.3)	11 (8.9)	
Pembrolizumab	10 (6.3)	10 (5.9)		8 (6.5)	6 (4.8)	
Toripalimab	10 (6.3)	18 (10.6)		10 (8.1)	11 (8.9)	
Durvalumab	4 (2.5)	3 (1.8)		4 (3.2)	3 (2.4)	
Targeted therapy^Δ^						
Lenvatinib	79 (49.7)	88 (51.8)	0.936	63 (50.8)	61 (49.2)	0.553
Sorafenib	15 (9.4)	20 (11.8)		14 (11.3)	11 (8.9)	
Donafenib	14 (8.8)	11 (6.5)		11 (8.9)	8 (6.5)	
Bevacizumab	26 (16.4)	25 (14.7)		20 (16.1)	20 (16.1)	
Apatinib	12 (7.5)	10 (5.9)		8 (6.5)	10 (8.1)	
Anlotinib	9 (5.7)	10 (5.9)		5 (4.0)	10 (8.1)	
Regorafenib	4 (2.5)	6 (3.5)		3 (2.4)	4 (3.2)	

*Note:* Δ: Fisher's exact test, others used *χ* ^2^ test.

Abbreviations: ALT, alanine aminotransferase; BCLC, barcelona‐clinic liver cancer; ECOG, eastern cooperative oncology group; PLT, platelet count.

### 3.2 Impact of LLV on Treatment Efficacy and Survival

In each analysis stage, the MVR group consistently showed higher metrics than the LLV group. Before PSM, the MVR group had a higher partial response (PR) and disease control rates (DCR), with PR at 36.5% versus 21.2% (*p* = 0.002) and DCR at 92.5% versus 78.8% (*p* < 0.001). Median overall survival (mOS) was also significantly longer in the MVR group, at 40.0 months (95% CI: 34.2–45.8), compared to 22.8 months (95% CI: 17.6–28.1) in the LLV group (*p* < 0.001).

**FIGURE 2:**
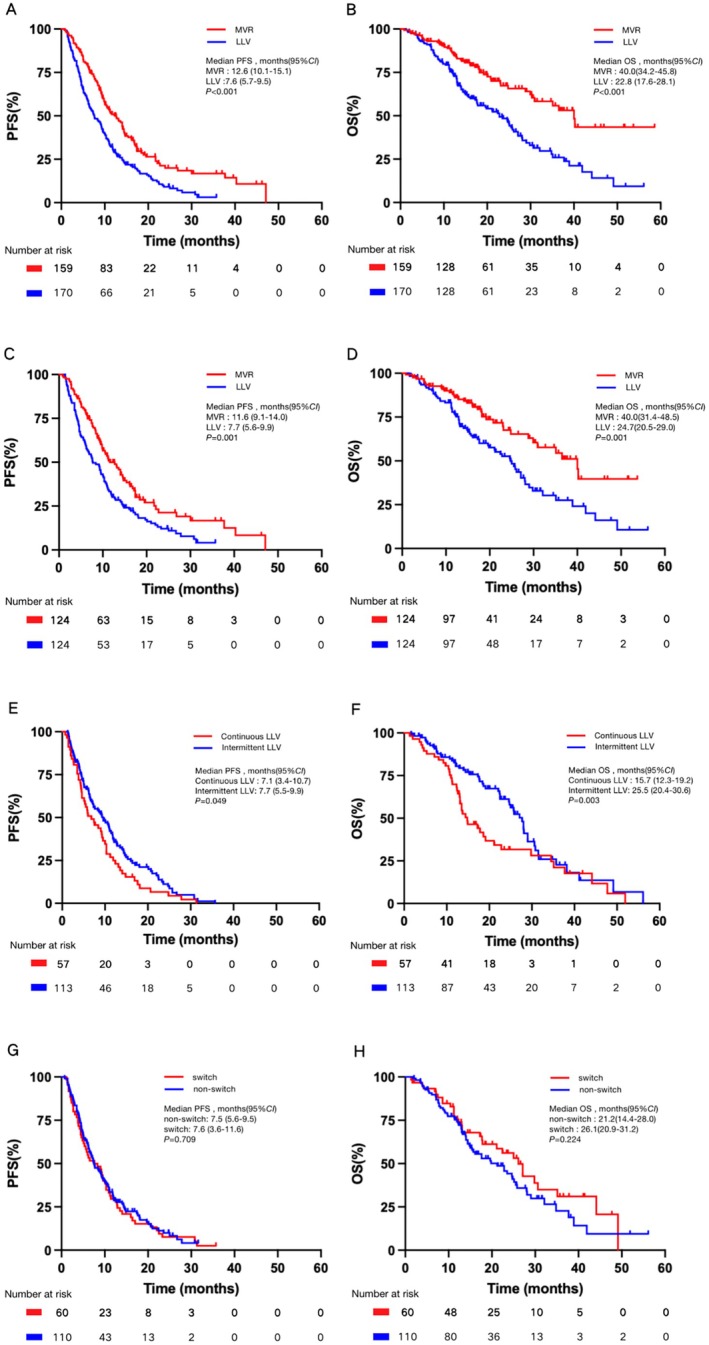
Kaplan–Meier curves for progression‐free survival and overall survival. (A) PFS curves for the patients before PSM. (B) OS curves for the patients before PSM. (C) PFS curves for the patients after PSM. (D) OS curves for the patients after PSM. (E) PFS curves for the patients with continuous LLV or intermittent LLV. (F) OS curves for the patients with continuous LLV or intermittent LLV. (G) PFS curves for the patients with adjusted treatment or continuation of original treatment. (H) OS curves for the patients with adjusted treatment or continuation of original treatment. CI, confidence interval.

After PSM, the MVR group maintained superior PR and DCR, with PR at 37.1% versus 21.0% (*p* = 0.005) and DCR at 91.9% versus 79.0% (*p* = 0.004) (Table 2). Median progression‐free survival (mPFS) was also longer for the MVR group, at 11.6 months (95% CI: 9.1–14.0) versus 7.7 months (95% CI: 5.6–9.9) in the LLV group (*p* = 0.001) (Figure 2).

**TABLE 2:** Tumour response.

Tumour response, *n* (%)	Before PSM			After PSM		
	MVR	LLV	*p*	MVR	LLV	*p*
Partial response (PR)	58 (36.5)	36 (21.2)		46 (37.1)	26 (21.0)	
Stable disease (SD)	89 (56.0)	98 (57.6)		68 (54.8)	72 (58.1)	
Progressive disease (PD)	12 (7.5)	36 (21.2)		10 (8.1)	26 (21.0)	
ORR	58 (36.5)	36 (21.2)	0.002	46 (37.1)	26 (21.0)	0.005
DCR	147 (92.5)	134 (78.8)	< 0.001	114 (91.9)	98 (79.0)	0.004

Abbrevations: DCR, disease control rate; ORR, objective response rate.

Following IPTW adjustment, mOS in the MVR group was 40.0 months (95% CI: 30.9‐NA) compared to 24.7 months (95% CI: 19.6–29.0) in the LLV group (*p* < 0.001), with mPFS at 13.3 months in the MVR group versus 8.57 months in the LLV group (*p* < 0.001) (Figure S2).

### 3.3 Impact of Continuous and Intermittent LLV on Treatment Efficacy and Survival

In the LLV cohort, patients were categorised into continuous LLV (CLLV) and intermittent LLV (ILLV) groups. In the CLLV group, 8 patients (14.0%) achieved PR, 35 (61.4%) achieved stable disease (SD), and 14 (24.6%) experienced progressive disease (PD), resulting in an ORR of 14.0% and a DCR of 75.4%. In the ILLV group, 28 patients (24.8%) achieved PR, 63 (55.8%) achieved SD, and 22 (19.5%) experienced PD, resulting in an ORR of 24.8% and a DCR of 80.5%. Despite these similar ORR and DCR values (*p* > 0.05) (Table 3), significant differences were observed in mPFS and mOS. The mPFS was shorter in the CLLV group (7.1 months, 95% CI: 3.4–10.7) compared to the ILLV group (7.7 months, 95% CI: 5.5–9.9) (*p* = 0.049). Similarly, mOS was also shorter at 15.7 months (95% CI: 12.3–19.2) for the CLLV group and 25.5 months (95% CI: 20.4–30.6) for ILLV, showing a significant difference (*p* = 0.003) (Figure 2).

**TABLE 3:** Tumour responses for the patients with continuous LLV or intermittent LLV.

Tumour response, *n* (%)	Continuous LLV	Intermittent LLV	*p*
Partial response (PR)	8 (14.0)	28 (24.8)	
Stable disease (SD)	35 (61.4)	63 (55.8)	
Progressive disease (PD)	14 (24.6)	22 (19.5)	
ORR	8 (14.0)	28 (24.8)	0.106
DCR	43 (75.4)	91 (80.5)	0.443

Abbrevations: DCR, disease control rate; ORR, objective response rate.

### 3.4 Impact of Altering Antiviral Therapy Regimens on Treatment Efficacy and Survival

Among the 170 LLV patients, 110 remained on the same antiviral regimen (non‐switch group). In comparison, 60 patients either switched to another NA, combined two NAs, or combined an NA with interferon (switch group). ORR was 22.7% in the non‐switch group and 18.3% in the switch group (*p* = 0.503), while the DCR was 80.0% and 76.7%, respectively (*p* = 0.611) (Table 4). Changing the NA antiviral regimen did not significantly impact PFS or OS, with mPFS of 7.5 months in the non‐switch group and 7.6 months in the switch group (*p* = 0.709), and mOS of 21.2 months versus 26.1 months (*p* = 0.224) (Figure 2).

**TABLE 4:** Tumour responses for the patients with adjusted treatment or continuation of original treatment.

Tumour response, *n* (%)	Non‐Switch	Switch	*p*
Partial response (PR)	25 (22.7)	11 (18.3)	
Stable disease (SD)	63 (57.3)	35 (58.3)	
Progressive disease (PD)	22 (20.0)	14 (23.3)	
ORR	25 (22.7)	11 (18.3)	0.503
DCR	88 (80.0)	46 (76.7)	0.611

Abbrevations: DCR, disease control rate; ORR, objective response rate.

### 3.5 LLV Is an Independent Risk Factor Affecting Patient Survival

**FIGURE 3:**
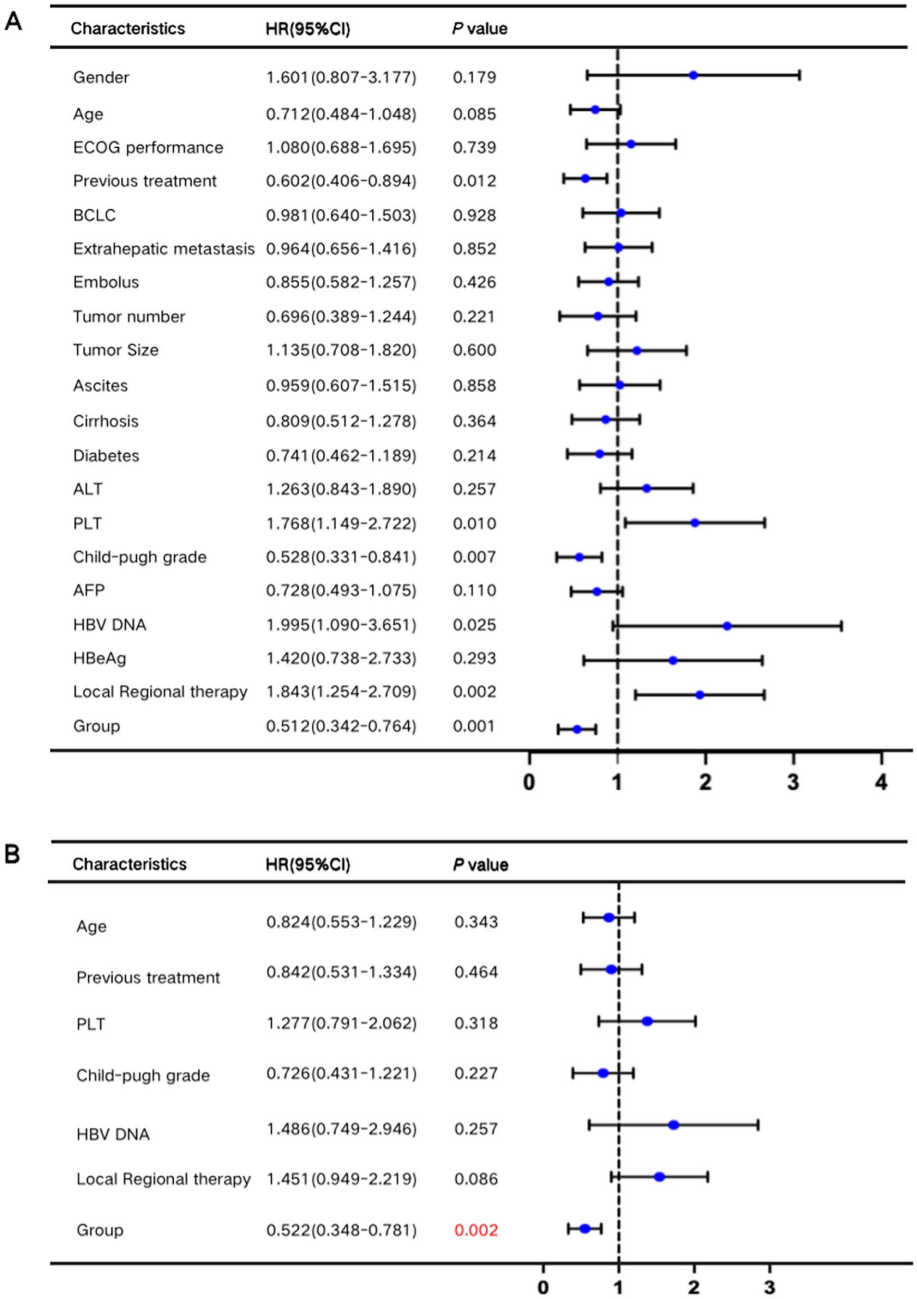
Univariate and multivariate Cox regression analyses of overall survival after PSM.

Univariate and multivariate Cox regression analyses were conducted to identify factors influencing OS. Univariate analysis highlighted several factors, including age, number of treatment lines, platelet count (PLT), Child‐Pugh classification, baseline HBV DNA levels, combined local therapy, and LLV status (*p* < 0.1). Multivariate analysis, however, identified LLV as the sole independent risk factor for OS (Figure 3).

### 3.6 Subgroup Analysis

The analysis of liver fibrosis and function indicators revealed that patients with higher APRI and FIB‐4 scores had poorer prognoses. Specifically, patients with APRI > 2 had a significantly shorter survival (14.0 months) compared to those with APRI ≤ 2 (38.9 months, *p* < 0.001). Those with FIB‐4 > 3.25 had a shorter survival (14.1 months) than those with FIB‐4 ≤ 3.25 (40.0 months, *p* < 0.001) (Figure S3).

Further intergroup analysis showed that the LLV group had more patients with severe liver fibrosis, as indicated by APRI scores > 2 and FIB‐4 scores > 3.25, compared to the MVR group (Figure S4). Additionally, liver function assessments showed that changes in ALBI grade and ICG15 retention rate from baseline to follow‐up were more pronounced in the LLV group, with the most significant increase observed in ICG15 (Figure S5). These findings suggest that the poorer prognosis in the LLV group may be attributed to progressive liver cirrhosis and diminished liver reserve.

### 3.7 Proteomic Analysis of Inflammation‐Related Proteins in LLV and MVR Groups

**FIGURE 4:**
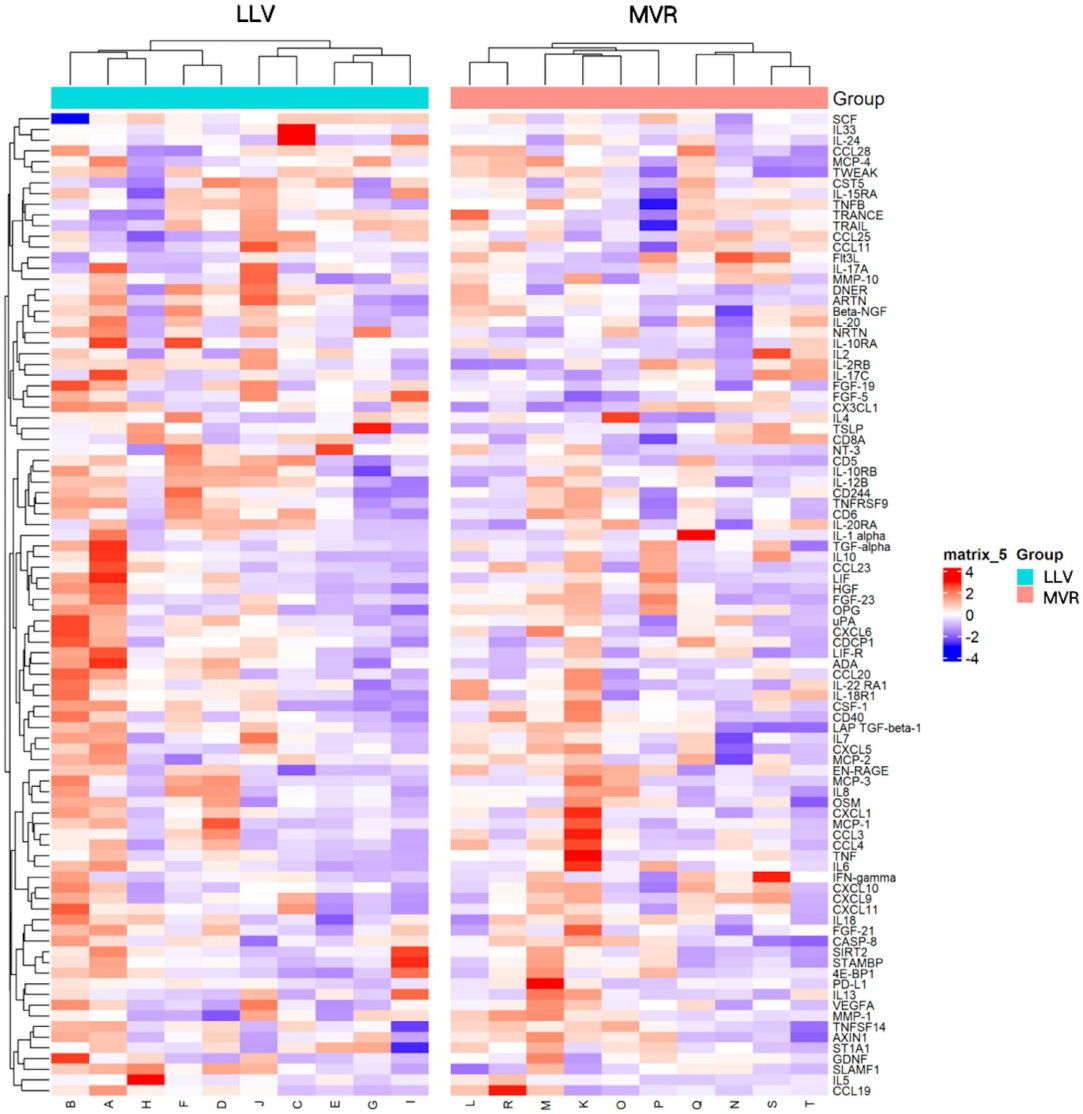
Protein clustering heatmap.

Proteomic profiling of serum samples from 10 patients (Table S2) in each group (LLV and MVR) identified significant differences in the expression of three inflammation‐related proteins: Fms‐related tyrosine kinase 3 ligand (Flt3L), signalling lymphocytic activation molecule family member 1 (SLAMF1) and fibroblast growth factor‐5 (FGF‐5). Flt3L was upregulated in the MVR group, while SLAMF1 and FGF‐5 were downregulated (Figure 4) (Table S3; Figures S6 and S7), with Flt3L exhibiting the most pronounced differential expression between the groups.

Functional enrichment analyses provided further insight into the biological implications of these differential proteins. Gene Ontology (GO) enrichment analysis indicated associations with immune‐related processes, such as phagosome formation, dendritic cell chemotaxis, and receptor‐ligand interactions. KEGG pathway analysis highlighted significant enrichment in the Ras, MAPK and PI3K‐Akt signalling pathways, while Reactome analysis underscored enrichment in the PI3K pathway, IRS‐mediated signalling and the insulin‐like growth factor receptor pathway (Figure S8).

## 4 Discussion

In this multicenter real‐world study, we assessed the impact of LLV on tumour response and survival in patients with HBV‐related uHCC treated with ICIs. Our findings demonstrated that LLV is associated with significantly lower ORR and DCR compared to patients maintaining a virological response (21.2% vs. 36.5%, *p* = 0.002; 78.8% vs. 92.5%, *p* < 0.001), as well as shorter mPFS and mOS (7.6 months and 22.8 months, respectively) compared to 12.6 months and 40.0 months in the MVR patients (*p* < 0.001). These differences persisted after PSM and IPTW adjustments. These results suggested that LLV may serve as a negative prognostic factor in ICIs‐based therapy for HBV‐related HCC.

This study also provided insights into underlying molecular mechanisms through proteomic analysis, revealing that LLV patients had significantly different levels of three inflammation‐related proteins—Flt3L, SLAMF1, and FGF‐5—compared to MVR patients. The downregulation of Flt3L, known for enhancing dendritic cell abundance and immune activation, may partly explain the poorer response to ICIs in LLV patients. Flt3L has shown promise as a biomarker of ICI responsiveness in other cancers, such as melanoma, by promoting conventional type 1 dendritic cell (cDC1) populations associated with anti‐tumour immunity [14, 15, 16, 17]. Conversely, SLAMF1 and FGF‐5, whose levels were elevated in LLV, are implicated in immune regulation and malignancy progression, possibly contributing to lower ICI efficacy in HCC [18, 19, 20, 21, 22, 23, 24].

Our study aligns with previous research indicating that LLV contributes to poorer clinical outcomes in HBV‐infected patients. For instance, Sinn et al. reported in a cohort of 875 treatment‐naive CHB patients on ETV that they found that LLV was associated with nearly double the risk of HCC development (HR 1.98, 95% CI 1.28–3.06, *p* = 0.002), identifying LLV and cirrhosis as independent risk factors for HCC progression [11]. Similarly, another study in China demonstrated a higher 5‐ and 10‐year incidence of end‐stage liver disease, including decompensated cirrhosis and HCC, in LLV patients compared to MVR patients (*p* < 0.050), with these trends persisting even after propensity score matching [12]. However, research focusing on the impact of LLV on HBV‐related HCC remains limited. Our study addresses this gap by examining LLV's effect on ICIs in HCC patients.

The underlying mechanisms by which LLV negatively affects ICIs efficacy may involve both hepatic and immunological factors. Persistent low‐level HBV replication can sustain chronic hepatic inflammation and promote fibrosis progression, leading to advanced cirrhosis and reduced liver reserve capacity [25]. Our subgroup analyses revealed that patients with LLV had higher APRI and FIB‐4 scores, indicating more severe liver fibrosis. Additionally, liver function assessments showed greater deterioration in ALBI grade and ICG15 among LLV patients during treatment, suggesting that progressive liver dysfunction may contribute to poorer outcomes.

Our proteomic analysis provided further insights into potential immunological mechanisms. We identified significant differences in the expression of three inflammation‐related proteins—Flt3L, SLAMF1 and FGF‐5—between LLV and MVR patients.

Notably, Flt3L was significantly downregulated in LLV patients. Flt3L plays a crucial role in the development and homeostasis of cDC1, which are essential for antigen presentation and the activation of cytotoxic T lymphocytes [14, 15, 16]. Reduced levels of Flt3L may impair dendritic cell function, leading to diminished anti‐tumour immune responses and decreased efficacy of ICIs. In other malignancies, higher Flt3L levels have been associated with better responses to ICIs, highlighting its potential as a biomarker for immunotherapy responsiveness [15, 17].

SLAMF1 was upregulated in LLV patients. SLAMF1 is involved in immune cell signalling and has been implicated in modulating immune responses in chronic infections and malignancies [18, 19, 20]. Elevated SLAMF1 expression may contribute to immune dysregulation and tumour immune evasion by altering the function of T cells and other immune cells. FGF‐5, another protein elevated in LLV patients, is part of the fibroblast growth factor family and has been associated with tumour progression and poor prognosis in various cancers, including HCC [21, 22, 23, 24]. Increased FGF‐5 levels may promote oncogenic signalling pathways, such as the MAPK and PI3K‐Akt pathways identified in our KEGG pathway analysis, further contributing to tumour growth and resistance to immunotherapy.

Our findings underscore the importance of achieving and maintaining complete virological suppression in HBV‐related HCC patients undergoing ICIs therapy. The presence of LLV may indicate ongoing viral replication and reflect a state of chronic inflammation and immune dysfunction that compromises the effectiveness of immunotherapies. While changing antiviral regimens did not significantly improve outcomes in LLV patients within our study, this might be due to the advanced stage of liver disease or insufficient time for the new regimen to exert its effects. Future research should explore whether more aggressive antiviral strategies or combination therapies can enhance viral suppression and improve immunotherapeutic efficacy.

## 5 Limitations

Several limitations should be noted in this study. First, its retrospective design could introduce bias, although we used PSM and IPTW to balance baseline characteristics. Second, we did not analyse changes in HBV DNA levels among LLV patients who switched antiviral therapies, which may influence long‐term outcomes. Third, the proteomic analysis was conducted on a relatively small subset of patients, which may limit the generalisability of the findings. Finally, our study did not investigate the precise molecular mechanisms through which LLV affects ICIs treatment efficacy in HCC, suggesting areas for future mechanistic studies.

## 6 Conclusion

In summary, our study highlights that LLV in uHCC patients receiving ICIs‐based antitumour therapy is associated with significantly lower tumour response rates and shorter survival compared to patients with MVR. These findings suggest that LLV may serve as a negative prognostic marker and emphasise the importance of strict viral suppression in optimising immunotherapy outcomes for HBV‐related HCC patients. Addressing LLV through effective antiviral strategies could potentially enhance the efficacy of ICIs and improve patient prognosis.

## Author Contributions

**Rong Li:** conceptualization, data curation, formal analysis, methodology, writing – original draft. **Wenli Li:** conceptualization, data curation, software, writing – original draft. **Qing Yang:** data curation, supervision, writing – review and editing. **Yujuan Guan:** data curation, supervision. **Yongru Chen:** data curation, writing – review and editing. **Peilin Zhu:** data curation, writing – review and editing. **Kaiyan Su:** data curation, writing – review and editing. **Qi Li:** data curation, methodology. **Xiaoyun Hu:** data curation, supervision. **Mengya Zang:** funding acquisition, methodology. **Miaoxian Zhao:** data curation. **Manhua Zhong:** data curation. **Jingquan Yan:** data curation. **Keli Yang:** data curation. **Wei zhu:** supervision, writing – review and editing. **Zhanzhou Lin:** software, writing – review and editing. **Guosheng Yuan:** funding acquisition, software, supervision, writing – review and editing. **Jinzhang Chen:** conceptualization, funding acquisition, methodology, supervision, writing – review and editing.

## Ethics Statement

The study was approved by the Southern Hospital Ethics Committee (Approval No. NFEC‐2023‐593).

## Consent

All patients signed informed consents before they received any treatment.

## Conflicts of Interest

The authors declare no conflicts of interest.

## Supporting information


Data S1.

## Data Availability

The data that support the findings of this study are available from the corresponding author upon reasonable request.
