# Supplementary material for: Low‐Level Viremia Impairs Efficacy of Immune Checkpoint Inhibitors in Unresectable Hepatocellular Carcinoma
Source: Liver Int. 2025 Mar 13;45(4):e70066. doi: 10.1111/liv.70066 (PMC11904444; doi:10.1111/liv.70066)
Supplement: Supplementary file 1 — Data S1. [file LIV-45-0-s001.docx]

Supplementary Table 1. Patient baseline characteristics before and after applying IPTW

|  |  | Before IPTW | |  |  | After IPTW | |  |  |  |
| --- | --- | --- | --- | --- | --- | --- | --- | --- | --- | --- |
| Characteristics | | MVR | LLV | P value | SMD | MVR | | LLV | P value | SMD |
|  |  | 159 | 170 |  |  | 339 | | 324 |  |  |
| Sex (%) | Female | 12 (7.5) | 13 (7.6) | 1 | 0.004 | 20.0 (5.9) | 22.0 (6.8) | | 0.748 | 0.034 |
|  | Male | 147 (92.5) | 157 (92.4) |  |  | 319.0 (94.1) | | 302.0 (93.2) | |  |
| ECOG PS (%) | 0 | 122 (76.7) | 122 (71.8) | 0.16 | 0.215 | 245.0 (71.8) | | 237.0 (73.1) | 0.811 | 0.085 |
|  | 1 | 36 (22.6) | 42 (24.7) |  |  | 90.0 (27.0) | | 80.0 (24.7) | |  |
|  | 2 | 1 (0.6) | 6 (3.5) |  |  | 4.0 (1.2) | | 7.0 (2.2) |  |  |
| BCLC (%) | B | 56 (35.2) | 40 (23.5) | 0.027 | 0.259 | 91.0 (26.8) | | 90.0 (27.8) | 0.880 | 0.018 |
|  | C | 103 (64.8) | 130 (76.5) |  |  | 248.0(73.2) | | 234.0 (72.2) | |  |
| Extrahepatic Metastasis (%) | Absent | 94 (59.1) | 80 (47.1) | 0.038 | 0.243 | 171.0 (50.4) | | 168.0 (51.9) | 0.799 | 0.032 |
|  | Present | 65 (40.9) | 90 (52.9) |  |  | 168.0 (49.6) | | 156.0 (48.1) | |  |
| Embolus (%) | Absent | 95 (59.7) | 92 (54.1) | 0.358 | 0.114 | 177.0 (51.2) | | 184.0 (56.8) | 0.441 | 0.096 |
|  | Present | 64 (40.3) | 78 (45.9) |  |  | 162.0 (48.8) | | 140.0 (43.2) | |  |
| Number of tumors (%) | ＜2 | 25 (15.7) | 33 (19.4) | 0.464 | 0.097 | 79.0 (23.3) | | 63.0 (19.4) | 0.505 | 0.092 |
|  | ≥2 | 134 (84.3) | 137 (80.6) |  |  | 260.0 (76.7) | | 261.0 (80.6) | |  |
| Cirrhosis (%) | Absent | 33 (20.8) | 38 (22.4) | 0.827 | 0.039 | 71.0 (20.9) | | 70.0 (21.7) | 0.910 | 0.014 |
|  | Present | 126 (79.2) | 132 (77.6) |  |  | 268.0 (79.1) | | 254.0 (78.3) | |  |
| Tumor diameter (%) | <10 | 128 (80.5) | 123 (72.4) | 0.108 | 0.193 | 238.0 (70.2) | | 241.0 (74.3) | 0.479 | 0.095 |
|  | ≥10 | 31 (19.5) | 47 (27.6) |  |  | 101.0 (29.8) | | 83.0 (25.7) | |  |
| Child Pugh grade (%) | A | 128 (80.5) | 139 (81.8) | 0.88 | 0.032 | 280.0 (82.6) | | 265.0 (81.8) | 0.872 | 0.019 |
|  | B | 31 (19.5) | 31 (18.2) |  |  | 59.0 (17.4) | | 59.0 (18.2) | |  |
| ALBI grade (%) | 1 | 50 (31.4) | 61 (35.9) | 0.609 | 0.11 | 116.0 (34.2) | | 109.0 (33.6) | 0.982 | 0.024 |
|  | 2 | 102 (64.2) | 100 (58.8) |  |  | 208.0 (61.4) | | 199.0 (61.4) | |  |
|  | 3 | 7 (4.4) | 9 (5.3) |  |  | 15.0 (4.4) | | 16.0 (4.9) |  |  |
| HBV DNA (%) | ＜2000IU/mL | 26 (16.4) | 74 (43.5) | <0.001 | 0.621 | 114.0 (34.6) | | 102.0 (31.5) | 0.719 | 0.048 |
|  | ≥2000IU/mL | 133 (83.6) | 96 (56.5) |  |  | 225.0 (65.4) | | 222.0 (68.5) | |  |
| AFP (%) | ≤100ng/ml | 88 (55.3) | 75 (44.1) | 0.054 | 0.226 | 156.0 (46.0) | | 156.0 (48.1) | 0.739 | 0.041 |
|  | ＞100ng/ml | 71 (44.7) | 95 (55.9) |  |  | 183.0 (54.0) | | 168.0 (51.9) | |  |
| History (%) | Absent | 79 (49.7) | 84 (49.4) | 1 | 0.005 | 187.0 (55.2) | | 167.0 (51.5) | 0.564 | 0.071 |
|  | Present | 80 (50.3) | 86 (50.6) |  |  | 152.0 (44.8) | | 157.0 (48.5) | |  |
| ALT (%) | ＜40U/L | 108 (67.9) | 97 (57.1) | 0.055 | 0.226 | 208.0 (61.4) | | 199.0 (61.4) | 0.967 | 0.005 |
|  | ≥40U/L | 51 (32.1) | 73 (42.9) |  |  | 131.0 (38.6) | | 125.0 (38.6) | |  |

Supplementary Table 2. Baseline characteristics of the patients included in the proteomics analysis

| Characteristics | MVR | LLV |
| --- | --- | --- |
| Patients | 10 | 10 |
| Male sex | 10(100.0) | 8(80.0) |
| Age≥60 years | 3(30.0) | 4(40.0) |
| ECOG performance |  |  |
| 0 | 7(70.0) | 10(100.0) |
| 1 | 3(30.0) | 0 |
| Previous treatment |  |  |
| no | 7(70.0) | 5(50.0) |
| yes | 3(30.0) | 5(50.0) |
| BCLC |  |  |
| B | 3(30.0) | 4(40.0) |
| C | 7(70.0) | 6(60.0) |
| Extrahepatic Metastasis | 3(30.0) | 3(30.0) |
| Embolus | 6(60.0) | 4(40.0) |
| Number of tumors ≥2 | 9(90.0) | 8(80.0) |
| Tumor diameter ≥10cm | 4(40.0) | 4(40.0) |
| Ascites | 4(40.0) | 2(20.0) |
| Cirrhosis | 7(70.0) | 8(80.0) |
| Diabetes | 1(10.0) | 0 |
| ALT levels ≥40U/L | 4(40.0) | 3(30.0) |
| Platelet count ≥100x10^9^/L | 9(90.0) | 9(90.0) |
| Child-Pugh grade |  |  |
| A | 10(100.0) | 8(80.0) |
| B | 0 | 2(20.0) |
| Serum AFP ≥400ng/ml | 2(20.0) | 5(50.0) |
| HBV DNA ≥2000IU/mL | 3(30.0) | 2(20.0) |
| HBeAg positive | 2(20.0) | 1(10.0) |

Supplementary Table 3. Information about three differentially expressed proteins

| Assay | Uniport | SYMBOL | ENTREZID | t | p | fdr | log2foldchange |
| --- | --- | --- | --- | --- | --- | --- | --- |
| Flt3L | P49771 | FLT3LG | 2323 | -2.4341 | 0.0310 | 0.9893 | 1.4167 |
| SLAMF1 | Q13291 | SLAMF1 | 6504 | 2.1772 | 0.0433 | 0.9893 | -0.4327 |
| FGF-5 | P12034 | FGF5 | 2250 | 2.1679 | 0.0446 | 0.9893 | -0.2962 |


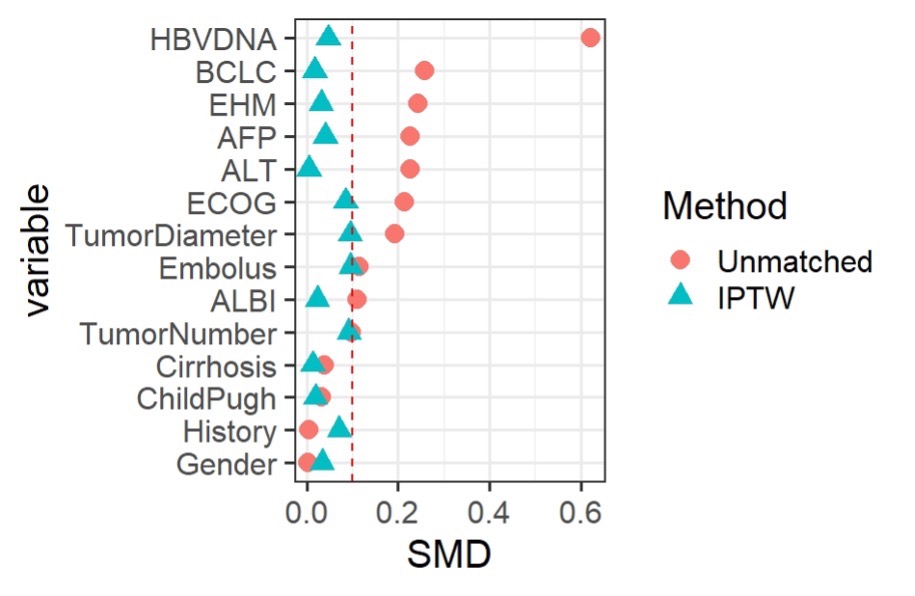


Supplementary Figure 1. Standardized Mean Differences (SMD) Before and After IPTW Matching


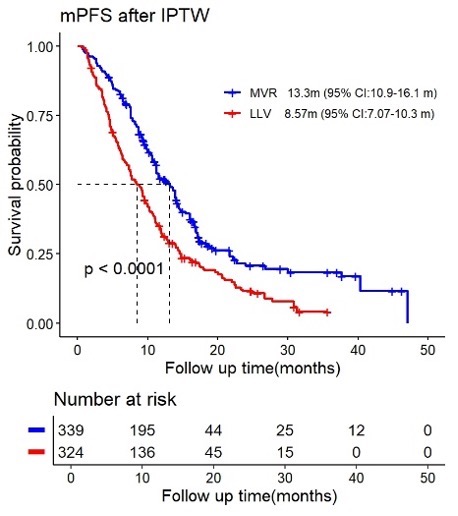

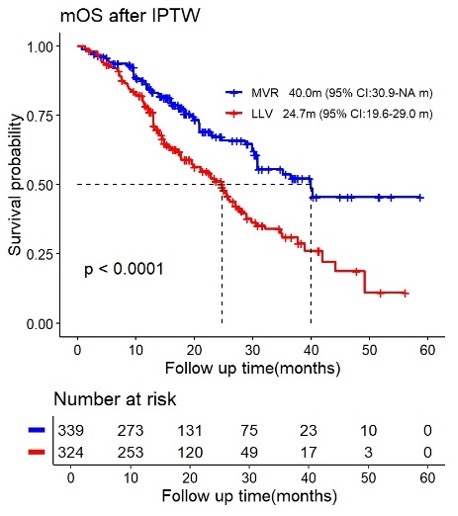


Supplementary Figure 2. Kaplan–Meier curves for progression-free survival and overall survival after IPTW.


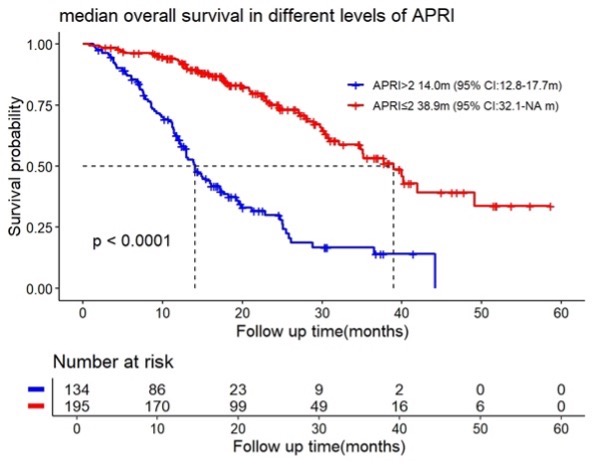

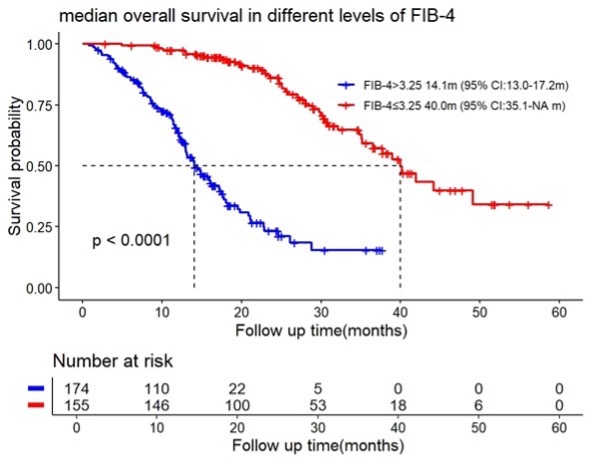


Supplementary Figure 3. Subgroup analysis.


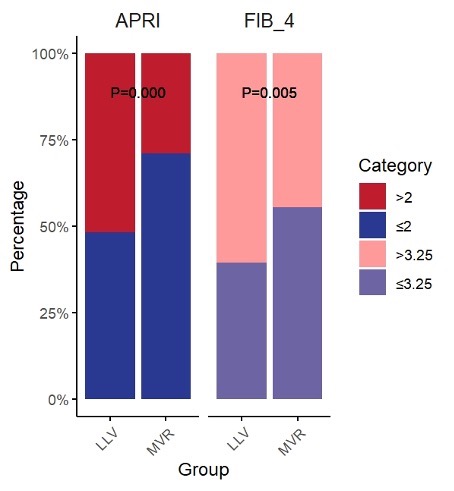


Supplementary Figure 4. Percentage comparison by group.


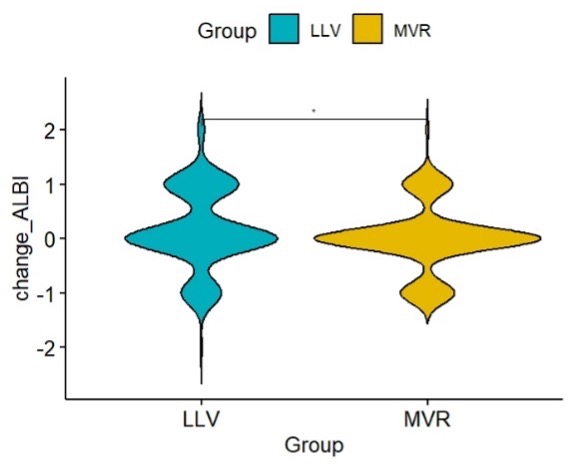

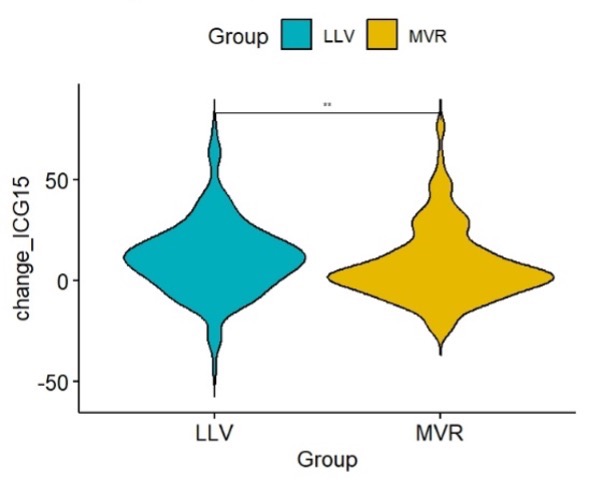


Supplementary Figure 5. Comparison of changes in ALBI and ICG15 between baseline and the last follow-up in two groups.


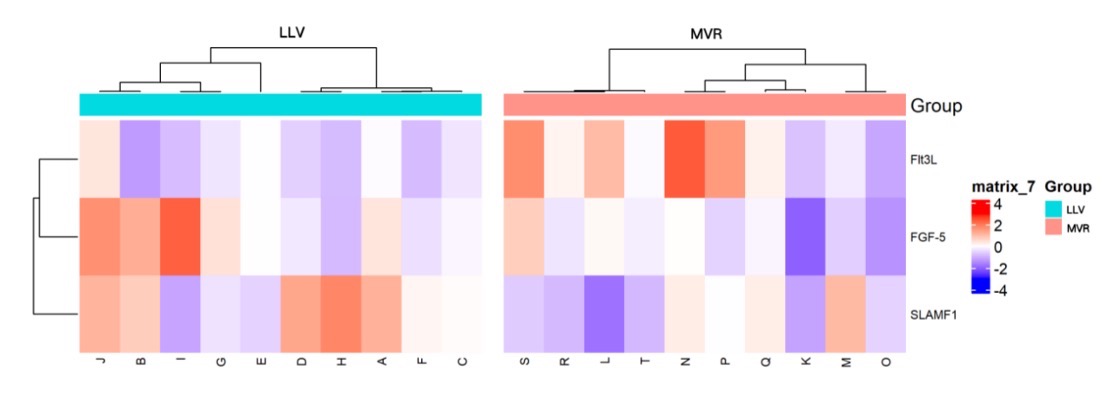


Supplementary Figure 6. Heat map of 3 differentially expressed proteins.


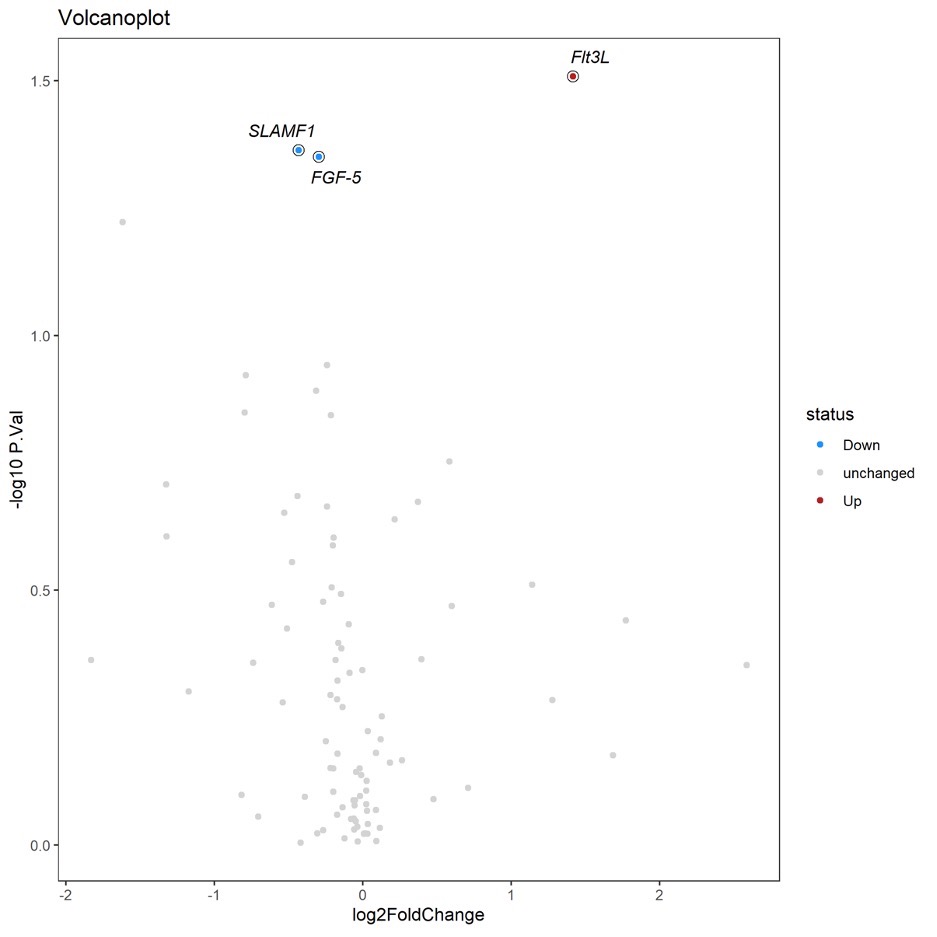


Supplementary Figure 7. Volcanic visualization of 3 differentially expressed proteins.


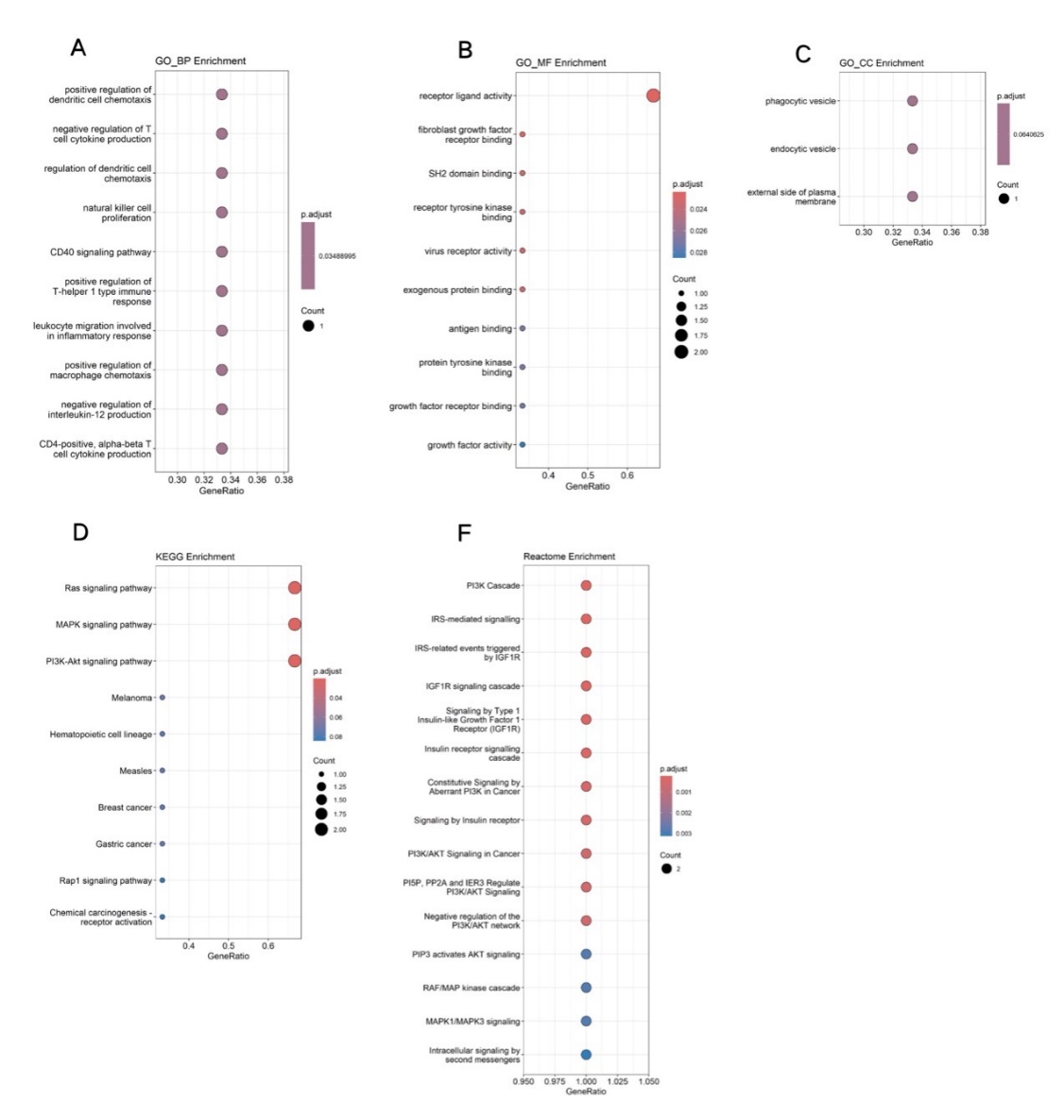


Supplementary Figure 8. Enrichment analysis of differentially expressed proteins. (A) GO(BP) enrichment analysis. (B) GO(MF) enrichment analysis. (C) GO(CC) enrichment analysis. (D) KEGG enrichment analysis. (E) Reactome enrichment analysis.
